# Determinants of morbidity and mortality following emergency abdominal surgery in children in low-income and middle-income countries

**DOI:** 10.1136/bmjgh-2016-000091

**Published:** 2016-12-12

**Authors:** GlobalSurg Collaborative, Adesoji O Ademuyiwa

**Affiliations:** Paediatric Surgery Unit, Department of Surgery, College of Medicine, Lagos University Teaching Hospital, University of Lagos, Lagos, Nigeria

## Abstract

**Background:**

Child health is a key priority on the global health agenda, yet the provision of essential and emergency surgery in children is patchy in resource-poor regions. This study was aimed to determine the mortality risk for emergency abdominal paediatric surgery in low-income countries globally.

**Methods:**

Multicentre, international, prospective, cohort study. Self-selected surgical units performing emergency abdominal surgery submitted prespecified data for consecutive children aged <16 years during a 2-week period between July and December 2014. The United Nation's Human Development Index (HDI) was used to stratify countries. The main outcome measure was 30-day postoperative mortality, analysed by multilevel logistic regression.

**Results:**

This study included 1409 patients from 253 centres in 43 countries; 282 children were under 2 years of age. Among them, 265 (18.8%) were from low-HDI, 450 (31.9%) from middle-HDI and 694 (49.3%) from high-HDI countries. The most common operations performed were appendectomy, small bowel resection, pyloromyotomy and correction of intussusception. After adjustment for patient and hospital risk factors, child mortality at 30 days was significantly higher in low-HDI (adjusted OR 7.14 (95% CI 2.52 to 20.23), p<0.001) and middle-HDI (4.42 (1.44 to 13.56), p=0.009) countries compared with high-HDI countries, translating to 40 excess deaths per 1000 procedures performed.

**Conclusions:**

Adjusted mortality in children following emergency abdominal surgery may be as high as 7 times greater in low-HDI and middle-HDI countries compared with high-HDI countries. Effective provision of emergency essential surgery should be a key priority for global child health agendas.

**Trial registration number:**

NCT02179112; Pre-results.

**Key questions**

**What is already known about this topic?**

- There are little prospective data describing the outcomes of paediatric surgery in low-resource settings.
- Emergency surgery is associated with more deaths and complications than elective surgery, but most studies carried out until now are in adults.

**What are the new findings?**

- After accounting for differences in case mix, the odds of death after emergency abdominal surgery could be as high as seven times greater in low-income countries compared with high-income countries.

**Recommendations for policy**

- The provision of effective essential surgery should be a key priority for global child health agendas and has significant potential to impact on the global burden of disease.

## Introduction

Little data are available addressing the safety profile and risk factors affecting morbidity and mortality in children undergoing surgery globally. Most studies have been in adults and almost invariably were performed in high-resource countries.^1–3^ Although it is estimated that about 234 million surgical procedures are performed annually worldwide, the percentage of these involving children remains unknown.^4^

Studies from low- and middle-income countries (LMICs) have shown that in the neonatal period, mortality is associated with sepsis, multiple exposures to anaesthesia (reoperation), postoperative bleeding and complex congenital anomalies.^5–8^ Other risk factors include non-availability of trained personnel, delayed presentation, childbirth outside a hospital and financial constraints of the caregivers.^9–11^

Emergency surgery generally carries a higher morbidity and mortality compared with elective procedures.^12^ ^13^ An estimated 33 000 emergency laparotomies in all ages are performed annually in the UK with a 15–20% mortality, which is 10-fold higher than that of elective cardiac surgery.^14^ Reasons for this high mortality are multifactorial; as well as patient-related factors, these may include staffing issues, access to operating theatres or access to diagnostic investigations.^14^ Unfortunately, most of these evidences have been derived from adult populations.

To date, no prospective, multicentre, international investigation has evaluated the determinants of morbidity or mortality after emergency abdominal surgery in children on a global scale. The aim of the current study was to evaluate the mortality and morbidity of emergency abdominal surgery in children across countries of different human development indices (HDIs).

## Methods

### Study design

This was a cohort study of children under the age of 16 years recruited from multiple hospitals performing emergency abdominal surgery. Predefined data items were collected according to a previously published protocol (ClinicalTrials.gov identifier: NCT02179112)^15^ using the Research Electronic Data Capture (REDCap) which is an online data capture system.^16^ While the UK National Health Service Research Ethics review considered this study exempt from formal research registration (South East Scotland Research Ethics Service, reference: NR/1404AB12), individual centres obtained their own audit, ethical or institutional approval as appropriate.

The collaborative model used has previously been described elsewhere.^17^ Investigators from self-selected surgical units identified consecutive patients within 2-week time intervals between 1 July 2014 and 31 December 2014. An open invitation for participation was disseminated through social media, personal contacts, email to authors of published emergency surgery studies and national/international surgical organisations. Short intensive data collection allowed surgical teams within these units to contribute meaningful numbers of patients without requiring additional resources. Multiple 2-week data collection periods within institutions was allowed.

### Patients and procedures

Any hospital performing emergency abdominal surgery, which included paediatric patients, could choose to be included (self-selecting). Consecutive patients under age of 16 years undergoing emergency abdominal surgery during a chosen 2-week period between 1 July 2014 and 31 December 2014 were included. Emergency abdominal surgery was defined as any unplanned, non-elective operation, including reoperation after a previous procedure. Abdominal surgery was defined as any open, laparoscopic or laparoscopic-converted procedure that entered the peritoneal cavity. Elective (planned) or semielective procedures (where a patient initially admitted as an emergency was then discharged from hospital and readmitted at a later time for surgery) were excluded.

### Data

Data were selected to be objective, standardised, easily transcribed and internationally relevant, in order to maximise record completion and accuracy. Recruited patients were followed up to day 30 after surgery or for the length of their inpatient stay where follow-up was not feasible. Records were uploaded by local investigators to the secure online REDCap website. The lead investigator at each site validated all cases prior to data submission. The submitted data were then checked centrally and where missing data were identified, the local lead investigator was contacted and requested to complete the record. Once vetted, the record was accepted into the data set for analysis.

### Outcome measures

The primary outcome measure was 30-day postoperative mortality, defined as the number of patients in the cohort who died within 30 days of surgery.^18^ In the event where 30-day follow-up was unavailable, outcome status at the point of discharge from hospital was recorded. A ‘30-day postoperative mortality/death during hospital stay’, is shortened to ‘30-day mortality’ to aid readability. The secondary outcome measures were 24-hour mortality, major and minor complication, and surgical site infection (SSI). Complications were defined on the Clavien-Dindo scale:^19^ minor complications as grade I/II (any deviation from the normal postoperative course with or without a need for pharmacological treatment but without requirement for surgical, endoscopic and radiological interventions or critical care admission); reintervention as grade III (surgical, endoscopic or radiological reintervention, without requirement for critical care admission); and major complication as grade IV (complication requiring critical care admission).

### Statistical analysis

The lack of pre-existing literature data in this subject meant that an a priori sample size determination was rendered difficult by unknown factors such as the effect of clustering and variation in mortality by diagnosis. Variation across different international health settings was assessed by stratifying participating centres by country into three tertiles according to the Human Development Index (HDI) rank. This is a composite statistic of life expectancy, education and income indices published by the United Nations (http://hdr.undp.org/en/statistics). Differences between HDI tertiles were tested with the Pearson χ^2^ test and Kruskal-Wallis test for categorical and continuous variables, respectively.

Fixed effect binary logistic regression models were explored, and the variables determined to be statistically and clinically important were entered into full multivariable models. Final full model choice was guided by the Akaike information criterion (AIC). Hierarchical multivariable logistic regression models (random intercept) were constructed with country as the first level and patients as the second level. HDI tertile and other explanatory variables were included as fixed effects. Other than HDI tertile, all fixed effects were considered at the level of the patient. Coefficients are expressed as ORs with CIs and p values derived from percentiles of 10 000 bootstrap replications. Level 1 and 2 model residuals were checked and first-order interactions were tested. Goodness of model fit is reported with the Hosmer and Lemeshow test, and predictive ability described by area under the receiver operating characteristic (ROC) curve (c-statistic). All analyses were undertaken using the R Foundation Statistical Programme (R 3.1.1).

## Results

### Patients

**Figure 1:**
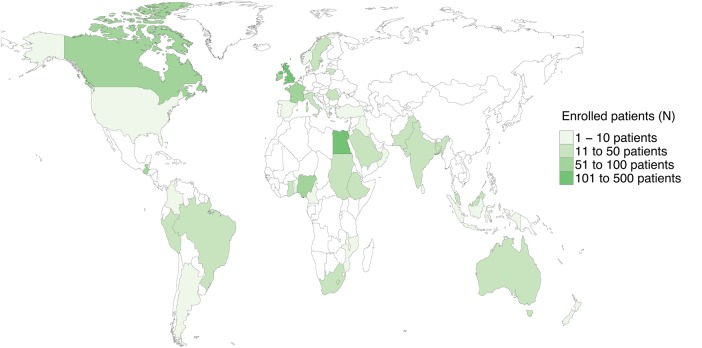
World map showing participating countries and number of enrolled patients.

A total of 1409 patients aged under 16 years, from 253 centres in 43 countries, were included in this study (figure 1). At the time of operation, 282 (20.0%) were under the age of 2 years. Of all children, 694 (49.3%) were from high-HDI, 450 (31.9%) from middle-HDI and 265 (18.8%) from low-HDI groups. There were slightly more males than females in all HDI groups (table 1) (55.9% in high-HDI, 61.1% in middle-HDI and 58.1% in low-HDI groups). Missing data rates were low, with one missing outcome for 24-hour mortality and one missing outcome for 30-day mortality. In 1140/1409 patients, 30-day outcomes, which otherwise represent status at discharge, were confirmed by direct patient contact (80.9%; high 572/694, 82.4%; middle 358/450, 79.6%; low 210/265, 79.2%; χ^2^ test, p=0.361).

**Table 1:** Patient characteristics

	HDI tertile			p Value
	High	Middle	Low	
Age in completed years				
Mean (SD)	8.9 (5.1)	9.1 (5.0)	7.0 (5.6)	<0.001
Gender				
Male	388 (55.9)	275 (61.1)	152 (57.4)	0.216
Female	306 (44.1)	175 (38.9)	113 (42.6)	
Missing	0 (0.0)	0 (0.0)	0 (0.0)	
ASA grade				
1	507 (73.1)	354 (78.7)	154 (58.1)	<0.001
2	105 (15.1)	65 (14.4)	58 (21.9)	
3	51 (7.3)	12 (2.7)	37 (14.0)	
4	23 (3.3)	6 (1.3)	12 (4.5)	
5	8 (1.2)	13 (2.9)	4 (1.5)	
Missing	0 (0.0)	0 (0.0)	0 (0.0)	
Surgical safety checklist used				
No, not available in this hospital	35 (5.0)	192 (42.7)	95 (35.8)	<0.001
No, but available in this hospital	6 (0.9)	39 (8.7)	74 (27.9)	
Yes	653 (94.1)	217 (48.2)	96 (36.2)	
Missing	0 (0.0)	2 (0.4)	0 (0.0)	
Perforated viscus				
No	596 (85.9)	399 (88.7)	190 (71.7)	<0.001
Yes	97 (14.0)	49 (10.9)	68 (25.7)	
Missing	1 (0.1)	2 (0.4)	7 (2.6)	
Prophylactic antibiotics				
No, not available	6 (0.9)	16 (3.6)	0 (0.0)	0.404*
No, but available	90 (13.0)	55 (12.2)	36 (13.6)	
Yes	598 (86.2)	377 (83.8)	228 (86.0)	
Missing	0 (0.0)	2 (0.4)	1 (0.4)	
Whole blood/products				
No, but available in this hospital	661 (95.2)	385 (85.6)	201 (75.8)	<0.001*
No, not available in this hospital	8 (1.2)	7 (1.6)	1 (0.4)	
Yes, whole blood	2 (0.3)	30 (6.7)	54 (20.4)	
Yes, blood products	23 (3.3)	26 (5.8)	9 (3.4)	
Missing	0 (0.0)	2 (0.4)	0 (0.0)	

*χ^2^ test is for yes versus no.

ASA, American Society of Anesthesiologists; HDI, Human Development Index.

### Demographics

Children undergoing emergency abdominal surgery in low-HDI countries had higher American Society of Anaesthesiologists (ASA) grades than children in middle-HDI or high-HDI groups (table 1). Furthermore, the WHO surgical safety checklist was employed prior to surgery in less than half of children undergoing emergency abdominal surgery from the low-HDI and middle-HDI groups compared with over 90% in the high-HDI group. At operation, 214/1406 (15.2%) of the children were found to have a perforated viscus, and this varied with HDI group (high 97/694, 14.0%; middle 49/450, 10.9%; low 68/265, 25.7%). Use of laparoscopy was widespread in high-HDI nations (341/694, 49.1%), whereas in middle-HDI (30/450, 6.7%) and low-HDI (8/257, 3.0%) settings, rates were much lower (p<0.001).

**Figure 2:**
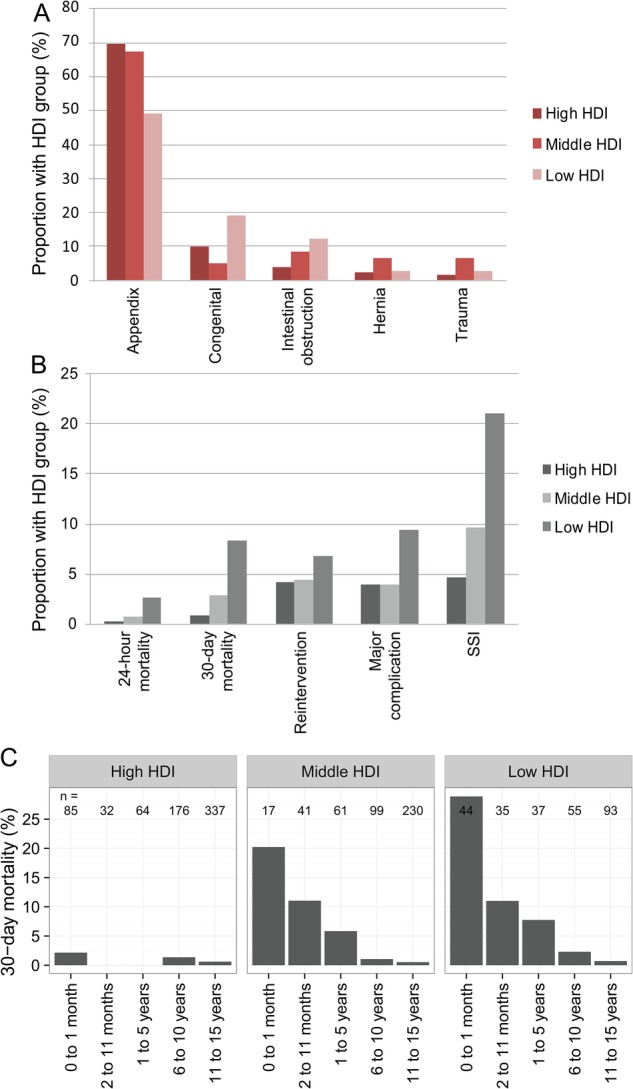
(A) Indications for emergency abdominal surgery in children across Human Developmental Index groups; (B) Surgical outcomes by Human Development Index group; (C) Adjusted 30-day mortality according to age groups. HDI, Human Developmental Index; SSI, surgical site infection.

Appendicitis was the most common indication for undergoing surgery across all groups, followed by congenital abnormalities, intussusception and hernia (figure 2A and online supplementary table S1). Emergency abdominal surgery for congenital abnormalities was significantly higher in low-HDI groups compared with middle-HDI and high-HDI groups (14.3% cf. 1.8% and 3.2%, respectively).

10.1136/bmjgh-2016-000091.supp1 supplementary tables

### Mortality

**Figure 3:**
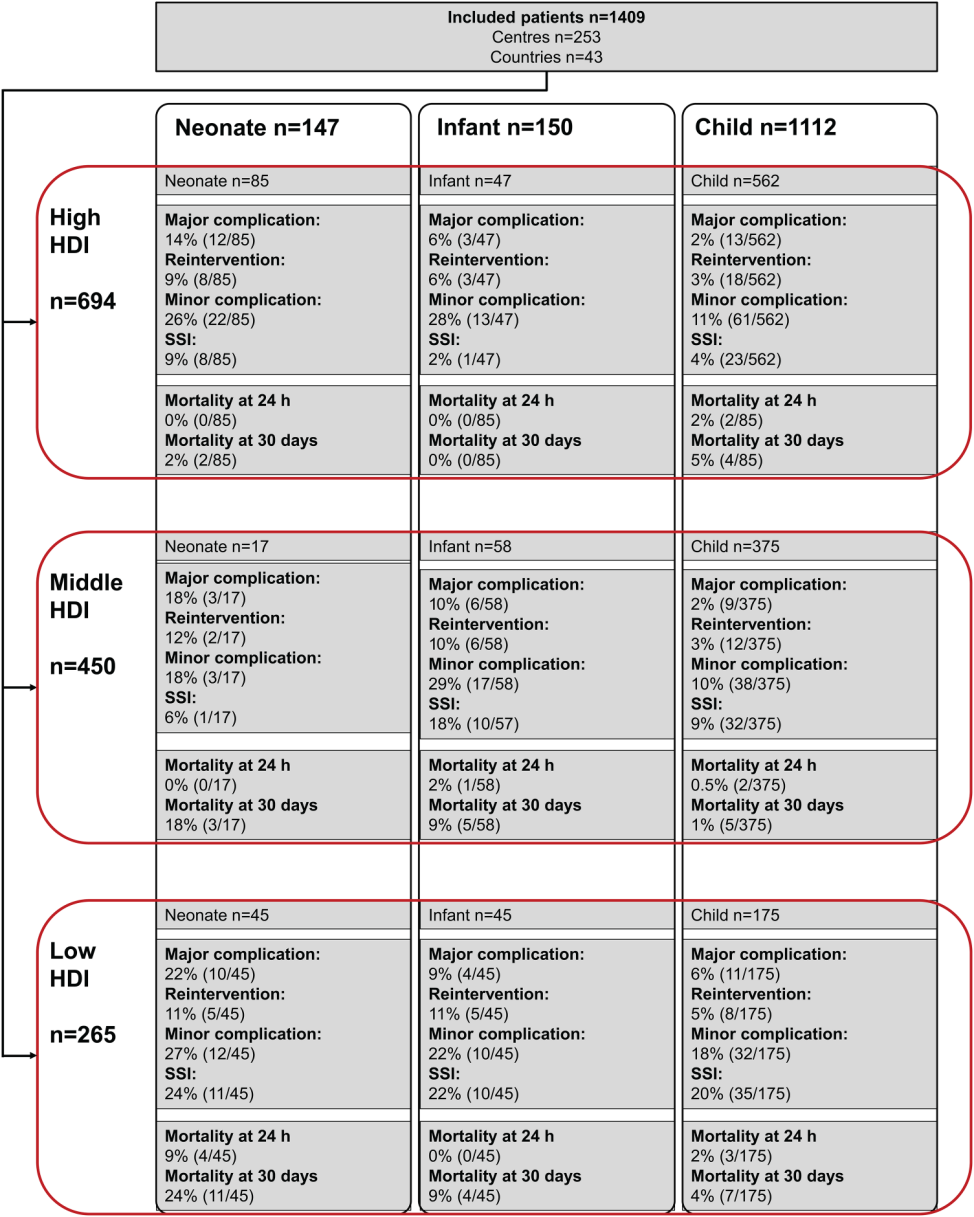
Patient complications and mortality profile according to Human Development Index. HDI, Human Developmental Index; SSI, surgical site infection.

Overall, 30-day mortality following surgery was 2.9% (n=41/1409) (figure 3). Of these deaths, 29.3% (n=12/41) occurred within 24 hours and 70.7% (n=29/41) between 24 hours and 30 days. Mortality varied significantly with HDI, with significantly higher proportions in low-HDI countries at 24 hours (0.3% in high-HDI, 0.7% in middle-HDI and 2.6% in low-HDI groups, p=0.005) and 30 days (0.9% in high-HDI, 2.9% in middle-HDI and 8.3% in low-HDI groups, p<0.001). Other associations with 24-hour and 30-day mortality in univariable analyses included neonatal age, >1 ASA grade and non-appendicitis procedures. Perforated viscus was significantly associated with 30-day mortality. An inversely proportional relationship is seen between 30-day mortality and age in all HDI groups even after adjustment in models (figure 2C).

In multilevel models, the association between low-HDI country, and 24-hour (OR 7.08, 95% CI 1.39 to 36.10, p=0.018) (table 2) and 30-day mortality (OR 7.79, 95% CI 2.96 to 20.48, p<0.001) (table 3) persisted. Middle-HDI country was associated with a 30-day mortality (OR 5.57, 95% CI 1.90 to 16.39, p=0.002) but not 24-hour mortality. A perforated viscus was significantly associated with increased 30-day mortality, whereas appendicitis was associated with lower 24-hour and 30-day mortality compared with other indications.

**Table 2:** Factors associated with 24-hour mortality

	Alive	Died	Univariate logistic regression OR (95% CI, p value)	Multilevel logistic regression OR (95% CI, p value)
HDI tertile				
High	692 (99.7)	2 (0.3)	–	–
Middle	446 (99.3)	3 (0.7)	2.33 (0.38 to 17.72, p=0.356)	3.71 (0.56 to 24.56, p=0.174)
Low	258 (97.4)	7 (2.6)	9.39 (2.25 to 63.28, p=0.005)	7.08 (1.39 to 36.10, p=0.018)
Age				
Child (>2 years <16 years)	1104 (99.4)	7 (0.6)	–	–
Infant (>1 month <2 years)	148 (99.3)	1 (0.7)	1.07 (0.06 to 6.05, p=0.953)	0.16 (0.02 to 1.45, p=0.102)
Neonate (≤1 month)	143 (97.3)	4 (2.7)	4.41 (1.14 to 14.79, p=0.019)	0.74 (0.16 to 3.33, p=0.694)
Gender				
Male	811 (99.5)	4 (0.5)	–	–
Female	585 (98.7)	8 (1.3)	2.77 (0.87 to 10.43, p=0.097)	3.47 (0.99 to 12.22, p=0.053)
ASA				
1	975 (99.8)	2 (0.2)	–	–
>1	421 (97.7)	10 (2.3)	11.58 (3.04 to 75.55, p=0.002)	5.22 (0.96 to 28.23, p=0.055)
Perforated viscus				
No	1177 (99.3)	8 (0.7)	–	–
Yes	209 (98.1)	4 (1.9)	2.82 (0.75 to 9.02, p=0.093)	1.57 (0.40 to 6.21, p=0.520)
Primary operation				
Non-appendicectomy	475 (97.7)	11 (2.3)	–	–
Appendicectomy	921 (99.9)	1 (0.1)	0.05 (0.00 to 0.24, p=0.003)	0.07 (0.01 to 0.59, p=0.015)

n=1398, AIC=120.2, c-statistic=0.922, H and L GOF=χ^2^=3.438, df=8, p value=0.904.

AIC, Akaike information criterion; ASA, American Society of Anesthesiologists; df, degree of freedom; H and L GOF, Hosmer-Lemeshow Goodness of fit; HDI, Human Development Index.

**Table 3:** Factors associated with 30-day mortality

	Alive	Died	Univariate logistic regression OR (95% CI, p value)	Multilevel logistic regression OR (95% CI, p value)
HDI tertile				
High	688 (99.1)	6 (0.9)	–	–
Middle	436 (97.1)	13 (2.9)	3.42 (1.34 to 9.79, p=0.013)	5.57 (1.90 to 16.39, p=0.002)
Low	243 (91.7)	22 (8.3)	10.38 (4.42 to 28.46, p<0.001)	7.79 (2.96 to 20.48, p<0.001)
Age				
Child (>2 years <16 years)	1095 (98.6)	16 (1.4)	–	–
Infant (>1 month<2 years)	140 (94.0)	9 (6.0)	4.40 (1.83 to 9.95, p=0.001)	0.91 (0.35 to 2.38, p=0.849)
Neonate (≤1 month)	131 (89.1)	16 (10.9)	8.36 (4.06 to 17.22, p<0.001)	2.27 (0.92 to 5.62, p=0.075)
Gender				
Male	794 (97.4)	21 (2.6)	–	–
Female	573 (96.6)	20 (3.4)	1.32 (0.70 to 2.47, p=0.382)	1.98 (1.00 to 3.94, p=0.051)
ASA				
1	964 (98.7)	13 (1.3)	–	–
>1	403 (93.5)	28 (6.5)	5.15 (2.69 to 10.37, p<0.001)	1.47 (0.67 to 3.25, p=0.337)
Perforated viscus				
No	1157 (97.6)	28 (2.4)	–	–
Yes	200 (93.9)	13 (6.1)	2.69 (1.33 to 5.17, p=0.004)	2.63 (1.21 to 5.73, p=0.015)
Primary operation				
Non-appendicectomy	447 (92.0)	39 (8.0)	–	–
Appendicectomy	920 (99.8)	2 (0.2)	0.02 (0.00 to 0.08, p<0.001)	0.04 (0.01 to 0.18, p<0.001)

n=1398, AIC=282.7, c-statistic=0.902, H&L GOF=χ^2^=6.418, df=8, p value=0.601.

AIC, Akaike information criterion; ASA, American Society of Anesthesiologists; df, degree of freedom; H and L, Hosmer-Lemeshow Goodness of fit; HDI, Human Development Index.

An analysis of predicted excess deaths was performed using the final multilevel 30-day mortality model. Based on this model, if all children in low-HDI and middle-HDI countries were considered to have been in high-HDI countries but otherwise had the same characteristics, 29 lesser deaths are predicted (40 per 1000 procedures).

### Major complications and reintervention

The overall rate of major complications following emergency abdominal surgery was 7.2% (n=102/1409) (figure 2B and online supplementary table S2). Major complications were significantly more common in low-HDI countries (11.3%, 30/265) compared with middle-HDI and high-HDI countries (6.4%, 29/450 and 6.2% 43/694, respectively, p=0.017). The rate of reintervention across the HDI groups mirrors these complications rates (low 6.8%, middle 4.4%, high 4.2%, p=0.222; online supplementary table S3).

### Minor complications

Across all HDI groups, the minor complication rate (Clavien-Dindo I-II) was 14.8% (n=208). This varied across HDI groups, with higher rates in low-HDI countries (20.9%) compared with middle-HDI and high-HDI countries (13.1% and 13.8% respectively, p=0.010), but these differences did not persist in multivariable analysis (see online supplementary table S4).

### Surgical site infection

The overall SSI rate was 9.3% (n=131). This varied significantly across HDI groups (low 21.1%, middle 9.6%, high 4.6%, p<0.001, online supplementary table S5).

## Discussion

The main findings of this study are sevenfold and fourfold higher 30-day mortalities in low-HDI and middle-HDI countries, respectively, compared with high-HDI countries. These rates are considerably greater than the threefold higher mortality previously reported among adult patients in low-HDI countries and account for an excess 40 deaths per thousand procedures in low-HDI and middle-HDI compared with high-HDI countries in this study alone.^20^ The risk factors for this excess mortality are necessarily multifactorial, including a higher intestinal perforation rate, which may reflect delayed access to surgery and different patterns of disease.

The twofold higher rate of major and minor postoperative complications and the fivefold difference in SSIs are also noteworthy. Our study does not allow us to identify the main factors responsible for these differences, but other studies in the literature point out a variety of aetiological factors including sepsis, multiple exposure to anaesthesia in the neonatal period, postoperative bleeding, as well as complexity of congenital anomaly, delayed presentation, non-availability of trained personnel and financial constraints on the part of the caregivers.^5–11^ ^21^ While the overall commonest surgical procedure in children remains appendicectomy, other complex procedures for congenital anomalies and intestinal obstruction are commonly performed in children in resource-limited settings. The similarity in procedures performed across resource settings was not expected, but it does demonstrate the depth of training required by surgical personnel to be able to handle such complex cases. Minimal access surgery was infrequently used in low-HDI and middle-HDI countries, showing inequality in access to contemporary technology through lack of resources including training in use of such technology.^22^

The study was able to draw from a large and diverse patient population, spanning wide geographical and resource areas globally. Despite the convenience sampling employed, it offers a snapshot of essential paediatric surgery across the globe. The main body of data from the study highlights the differences in pathology, patient premorbid status, operative findings and outcomes based on HDI grouping. The higher ASA status of children requiring emergency abdominal surgery in low-HDI and middle-HDI countries settings is concerning, and it potentially reflects delayed access to care with the consequent negative impact on postoperative outcomes. Similarly, the percentage of perforated viscus encountered at surgery was also significantly higher in low-HDI and middle-HDI countries. The delay in access to care has been previously reported by studies from LMICs.^9–11^ This may account for the poor survival of neonates with severe congenital anomalies in these settings, such as intestinal atresia, abdominal wall defects and oesophageal atresia.^11^ ^23^ A study from Nigeria indicated that delayed intervention time >72 hours, neonatal age and severe postoperative complications are associated with higher mortality in paediatric surgical emergencies.^21^

This study has some limitations. Being based on convenience sampling of hospitals, the data collected may not be truly representative of other sites which may be more poorly resourced. Collection bias, however, may result in the true outcomes being even worse in LMICs, as the lowest resource sites would be less likely to participate. In addition, other factors such as availability of personnel, availability of complex anaesthetic and intensive care support, and delay time before surgery were not analysed in this study but may significantly impact on postoperative mortality. The current study has documented differences in surgical outcomes in children based on HDI groups, but has not explored in depth the reasons for these differences. This will form the agenda for future studies, together with outcome studies, focusing on elective essential surgical procedures in children.

The main conclusion of this study is that emergency abdominal surgery in children is associated with significantly worse outcomes in LMICs. The documentation provided by this study is essential to the process of scaling up surgical services for children in low-resource settings. Good surgical outcomes require a multitude of factors, including trained personnel, good facilities and surgical supplies, as well as prompt access to surgical care. Thus, any single intervention in this multifaceted system has a high likelihood of failing to fully address these complex issues. This relates to many well-meaning efforts from high-income countries (HICs) to assist surgically in resource-limited settings. For instance, temporary platforms in the form of ‘surgical safaris’, the provision of surgical equipment alone, or short-term training courses outside one's normal work setting will likely have little long-term impact.^24^ ^25^ The likeliest context in which broad systematic change can occur is likely that of a long-lasting institutional partnership. In such a context of relationship with mutual understanding and trust, appropriate change can be implemented in whichever areas are most needed, and progress can be monitored and evaluated.^26^

The recent global recognition of surgery as an essential healthcare component has provided a unique impetus for provision of essential surgical services, especially in LMICs.^27^ ^28^ The task ahead is a huge one, in terms of access to and quality of care. The current study has documented relatively poor outcomes of emergency abdominal surgery in children in low-HDI and middle-HDI countries. Such data are essential in guiding efforts to improve the surgical care of children globally and prioritise it in the global health agenda.
