# Correction: *Determinants of morbidity and mortality following emergency abdominal surgery in children in low-income and middleincome countries*

**DOI:** 10.1136/bmjgh-2016-000091corr1

**Published:** 2017-01-30

GlobalSurg Collaborative. Determinants of morbidity and mortality following emergency abdominal surgery in children in low-income and middleincome countries. *BMJ Global Health* 2016;1:e000091. In the Collaborators section ‘Ahmed Negeida’ should be ‘Ahmed Negida’ and ‘Mohamed Fadel’ should be ‘Mohamed F Zalabia’.

