# Supplementary material for: Determinants of morbidity and mortality following emergency abdominal surgery in children in low-income and middle-income countries
Source: BMJ Glob Health. 2016 Dec 12;1(4):e000091. doi: 10.1136/bmjgh-2016-000091 (PMC5321375; doi:10.1136/bmjgh-2016-000091)
Supplement: supplementary tables [file bmjgh-2016-000091supp001.pdf]

Supplementary data

Table S1: Diagnosis

	Human development index			
	High	Middle	Low	Total
Appendicitis	483 (69.6)	303 (67.3)	130 (49.1)	916 (65.0)
Congenital	69 (9.9)	22 (4.9)	51 (19.2)	142 (10.1)
Intestinal obstruction	27 (3.9)	38 (8.4)	32 (12.1)	97 (6.9)
Hernia	15 (2.2)	29 (6.4)	7 (2.6)	51 (3.6)
Trauma	11 (1.6)	30 (6.7)	7 (2.6)	48 (3.4)
No disease identified	23 (3.3)	7 (1.6)	3 (1.1)	33 (2.3)
Complication of previous surgical operation	12 (1.7)	6 (1.3)	6 (2.3)	24 (1.7)
Female reproductive	21 (3.0)	0 (0.0)	2 (0.8)	23 (1.6)
Infection	4 (0.6)	1 (0.2)	16 (6.0)	21 (1.5)
Colitis	11 (1.6)	4 (0.9)	0 (0.0)	15 (1.1)
Other diagnosis	9 (1.3)	2 (0.4)	2 (0.8)	13 (0.9)
Perforation of intestine	2 (0.3)	0 (0.0)	3 (1.1)	5 (0.4)
Acute pancreatitis	1 (0.1)	3 (0.7)	0 (0.0)	4 (0.3)
Adhesions	1 (0.1)	2 (0.4)	1 (0.4)	4 (0.3)
Foreign body in GI tract	1 (0.1)	2 (0.4)	0 (0.0)	3 (0.2)
Neoplasm	3 (0.4)	0 (0.0)	0 (0.0)	3 (0.2)
Peptic ulcer	1 (0.1)	0 (0.0)	1 (0.4)	2 (0.1)
Perforation of oesophagus	0 (0.0)	1 (0.2)	1 (0.4)	2 (0.1)
Cholelithiasis	0 (0.0)	0 (0.0)	1 (0.4)	1 (0.1)
Fistula of intestine	0 (0.0)	0 (0.0)	1 (0.4)	1 (0.1)
Peritonitis	0 (0.0)	0 (0.0)	1 (0.4)	1 (0.1)

Table S2: Major complication

		No	Yes	Univariate logistic regression Odds ratio (95% CI, p-value)	Multilevel logistic regression Odds ratio (95% CI, p-value)
HDI tertile	High	666 (96.0)	28 (4.0)		
	Middle	429 (96.0)	18 (4.0)	1.00 (0.54-1.81, p=0.995)	1.40 (0.58-3.39, p=0.457)
	Low	240 (90.6)	25 (9.4)	2.48 (1.41-4.34, p=0.001)	1.30 (0.61-2.76, p=0.500)
Age	Child (>2 y <16 y)	1077 (97.0)	33 (3.0)		
	Infant (>1 month < 2 y)	136 (91.3)	13 (8.7)	3.12 (1.55-5.94, p=0.001)	0.78 (0.35-1.72, p=0.532)
	Neonate (<= 1 month)	122 (83.0)	25 (17.0)	6.69 (3.82-11.59, p<0.001)	1.29 (0.63-2.67, p=0.484)
Gender	Male	772 (94.7)	43 (5.3)		
	Female	563 (95.3)	28 (4.7)	0.89 (0.54-1.45, p=0.649)	0.98 (0.56-1.70, p=0.931)
ASA	1	956 (98.0)	20 (2.0)		
	>1	379 (88.1)	51 (11.9)	6.43 (3.84-11.17, p<0.001)	2.41 (1.26-4.58, p=0.008)
Perforated viscus	No	1145 (96.8)	38 (3.2)		
	Yes	180 (84.5)	33 (15.5)	5.52 (3.36-9.03, p<0.001)	6.26 (3.45-11.36, p<0.001)
Primary operation	Non-appendicectomy	420 (86.8)	64 (13.2)		
	Appendicectomy	915 (99.2)	7 (0.8)	0.05 (0.02-0.10, p<0.001)	0.06 (0.02-0.15, p<0.001)

HDI = Human Development Index; ASA = American Society of Anesthesiologists; OR = odds ratio; CI = confidence interval. n = 1396, AIC = 421.3, c-statistic = 0.888, H&L GOF = X-squared = 7.1444, df = 8, p-value = 0.5211.

Table S3: Reintervention

		No	Yes	Univariate logistic regression Odds ratio (95% CI, p-value)	Multilevel logistic regression Odds ratio (95% CI, p-value)
HDI tertile	High	665 (95.8)	29 (4.2)		
	Middle	427 (95.5)	20 (4.5)	1.07 (0.59-1.91, p=0.810)	1.16 (0.62-2.17, p=0.648)
	Low	247 (93.2)	18 (6.8)	1.67 (0.90-3.04, p=0.097)	0.98 (0.51-1.88, p=0.954)
Age	Child (>2 y <16 y)	1072 (96.6)	38 (3.4)		
	Infant (>1 month < 2 y)	135 (90.6)	14 (9.4)	2.93 (1.50-5.42, p=0.001)	1.42 (0.65-3.12, p=0.382)
	Neonate (<= 1 month)	132 (89.8)	15 (10.2)	3.21 (1.67-5.87, p<0.001)	1.26 (0.57-2.80, p=0.574)
Gender	Male	775 (95.1)	40 (4.9)		
	Female	564 (95.4)	27 (4.6)	0.93 (0.56-1.52, p=0.768)	1.03 (0.61-1.75, p=0.909)
ASA	1	946 (96.9)	30 (3.1)		
	>1	393 (91.4)	37 (8.6)	2.97 (1.81-4.90, p<0.001)	1.44 (0.80-2.59, p=0.219)
Perforated viscus	No	1144 (96.7)	39 (3.3)		
	Yes	185 (86.9)	28 (13.1)	4.44 (2.65-7.37, p<0.001)	4.75 (2.73-8.26, p<0.001)
Primary operation	Non-appendicectomy	438 (90.5)	46 (9.5)		
	Appendicectomy	901 (97.7)	21 (2.3)	0.22 (0.13-0.37, p<0.001)	0.29 (0.14-0.56, p<0.001)

HDI = Human Development Index; ASA = American Society of Anesthesiologists; OR = odds ratio; CI = confidence interval. n = 1396, AIC = 490.1, c-statistic = 0.766, H&L GOF = X-squared = 4.1922, df = 8, p-value = 0.8394.

Table S4: Minor complication

		No	Yes	Univariate logistic regression Odds ratio (95% CI, p-value)	Multilevel logistic regression Odds ratio (95% CI, p-value)
HDI tertile	High	598 (86.2)	96 (13.8)		
	Middle	384 (86.9)	58 (13.1)	0.94 (0.66-1.33, p=0.733)	1.00 (0.69-1.45, p=0.995)
	Low	204 (79.1)	54 (20.9)	1.65 (1.13-2.38, p=0.008)	1.14 (0.77-1.69, p=0.508)
Age	Child (>2 y <16 y)	970 (88.1)	131 (11.9)		
	Infant (>1 month < 2 y)	107 (72.8)	40 (27.2)	2.77 (1.83-4.13, p<0.001)	1.74 (1.02-2.97, p=0.040)
	Neonate (<= 1 month)	109 (74.7)	37 (25.3)	2.51 (1.64-3.78, p<0.001)	1.35 (0.78-2.36, p=0.288)
Gender	Male	689 (85.0)	122 (15.0)		
	Female	497 (85.2)	86 (14.8)	0.98 (0.72-1.32, p=0.880)	1.08 (0.79-1.48, p=0.628)
ASA	1	865 (89.1)	106 (10.9)		
	>1	321 (75.9)	102 (24.1)	2.59 (1.92-3.50, p<0.001)	1.65 (1.16-2.35, p=0.005)
Perforated viscus	No	1028 (87.2)	151 (12.8)		
	Yes	155 (73.1)	57 (26.9)	2.50 (1.76-3.53, p<0.001)	2.50 (1.72-3.62, p<0.001)
Primary operation	Non-appendicectomy	363 (76.6)	111 (23.4)		
	Appendicectomy	823 (89.5)	97 (10.5)	0.39 (0.29-0.52, p<0.001)	0.61 (0.40-0.95, p=0.028)

HDI = Human Development Index; ASA = American Society of Anesthesiologists; OR = odds ratio; CI = confidence interval. n = 1391, AIC = 1113.9, c-statistic = 0.681, H&L GOF = X-squared = 10.465, df = 8, p-value = 0.2339.

Table S5: Surgical site infection

		No	Yes	Univariate logistic regression Odds ratio (95% CI, p-value)	Multilevel logistic regression Odds ratio (95% CI, p-value)
HDI tertile	High	662 (95.4)	32 (4.6)		
	Middle	401 (90.3)	43 (9.7)	2.22 (1.38-3.59, p=0.001)	2.34 (1.21-4.55, p=0.012)
	Low	209 (78.9)	56 (21.1)	5.54 (3.52-8.88, p<0.001)	4.30 (2.41-7.65, p<0.001)
Age	Child (>2 y <16 y)	1017 (91.9)	90 (8.1)		
	Infant (>1 month < 2 y)	128 (85.9)	21 (14.1)	1.85 (1.09-3.03, p=0.018)	0.89 (0.46-1.74, p=0.738)
	Neonate (<= 1 month)	127 (86.4)	20 (13.6)	1.78 (1.03-2.93, p=0.029)	0.97 (0.48-1.94, p=0.925)
Gender	Male	738 (90.8)	75 (9.2)		
	Female	534 (90.5)	56 (9.5)	1.03 (0.71-1.48, p=0.866)	1.20 (0.81-1.78, p=0.358)
ASA	1	909 (93.4)	64 (6.6)		
	>1	363 (84.4)	67 (15.6)	2.62 (1.82-3.78, p<0.001)	1.77 (1.13-2.79, p=0.013)
Perforated viscus	No	1099 (93.1)	82 (6.9)		
	Yes	165 (77.8)	47 (22.2)	3.82 (2.56-5.64, p<0.001)	3.37 (2.17-5.23, p<0.001)
Primary operation	Non-appendicectomy	413 (85.5)	70 (14.5)		
	Appendicectomy	859 (93.4)	61 (6.6)	0.42 (0.29-0.60, p<0.001)	0.60 (0.36-1.00, p=0.050)

HDI = Human Development Index; ASA = American Society of Anesthesiologists; OR = odds ratio; CI = confidence interval. n = 1393, AIC = 773.9, c-statistic = 0.740, H&L GOF = X-squared = 11.929, df = 8, p-value = 0.1544.
